# High frequency of cerebrospinal fluid autoantibodies in patients with seizures or epilepsies of unknown etiology

**DOI:** 10.3389/fneur.2023.1211812

**Published:** 2023-07-05

**Authors:** Paulina Schulz, Alva Lütt, Winfried Stöcker, Bianca Teegen, Martin Holtkamp, Harald Prüss

**Affiliations:** ^1^German Center for Neurodegenerative Diseases (DZNE) Berlin, Berlin, Germany; ^2^Department of Neurology and Experimental Neurology, Charité-Universitätsmedizin Berlin, Corporate Member of Freie Universität Berlin, Humboldt-Universität Berlin, Berlin, Germany; ^3^Psychiatric University Hospital Charité at St. Hedwig Hospital, Berlin, Germany; ^4^Department of Psychiatry and Neurosciences, CCM, Charité Universitätsmedizin Berlin, Corporate Member of Freie Universität and Humboldt-Universität zu Berlin, Berlin, Germany; ^5^Berlin Institute of Health at Charité—Universitätsmedizin Berlin, Berlin, Germany; ^6^Institute for Experimental Immunology, Lübeck, Germany; ^7^Epilepsy-Center Berlin-Brandenburg, Institute for Diagnostics of Epilepsy, Berlin, Germany

**Keywords:** cerebrospinal fluid, autoimmune, unknown etiology, seizure, autoantibodies, encephalitis, immunofluorescence, autoimmune-associated epilepsy

## Abstract

**Introduction:**

The increasing identification of specific autoantibodies against brain structures allows further refinement of the group of autoimmune-associated epilepsies and affects diagnostic and therapeutic algorithms. The early etiological allocation of a first seizure is particularly challenging, and the contribution of cerebrospinal fluid (CSF) analysis is not fully understood.

**Methods:**

In this retrospective study with a mean of 7.8 years follow-up involving 39 well-characterized patients with the initial diagnosis of new-onset seizure or epilepsy of unknown etiology and 24 controls, we determined the frequency of autoantibodies to brain proteins in CSF/serum pairs using cell-based assays and unbiased immunofluorescence staining of unfixed murine brain sections.

**Results:**

Autoantibodies were detected in the CSF of 30.8% of patients. Underlying antigens involved glial fibrillary acidic protein (GFAP) and N-methyl-D-aspartate (NMDA) receptors, but also a range of yet undetermined epitopes on neurons, glial and vascular cells. While antibody-positive patients had higher frequencies of cancer, they did not differ from antibody-negative patients with respect to seizure type, electroencephalography (EEG) and cranial magnetic resonance imaging (cMRI) findings, neuropsychiatric comorbidities or pre-existing autoimmune diseases. In 5.1% of patients with seizures or epilepsy of initially presumed unknown etiology, mostly CSF findings resulted in etiological reallocation as autoimmune-associated epilepy.

**Discussion:**

These findings strengthen the potential role for routine CSF analysis. Further studies are needed to understand the autoantibody contribution to etiologically unclear epilepsies, including determining the antigenic targets of underlying autoantibodies.

## Introduction

Seizures and epilepsy are common in autoimmune encephalitis (1) and can even represent the main complaints, which led to the clinical entity of “immune epilepsy” that has been added to the International League Against Epilepsy (ILAE) classification of the epilepsies in 2017 (2) as an etiologic subgroup categorized as “immune disorder in which seizures are a core symptom.” In 2020, the ILAE Autoimmunity and Inflammation Taskforce proposed conceptual definitions for two main diagnostic entities in this field: (a) acute symptomatic seizures secondary to autoimmune encephalitis, and (b) autoimmune-associated epilepsy, the latter of which suggests an enduring predisposition to seizures (3) and corresponds to “immune epilepsy” in the 2017 ILAE classification.

Research on the clinical phenotypes and the underlying disease mechanisms has become a major focus over the past decade (4–7). In many patients, well-established autoantibodies are detected in clinical routine, such as autoantibodies against NMDA receptors (NMDAR) or leucine-rich glioma inactivated protein 1 (LGl1) (8, 9). In antibody-positive patients, new immunotherapeutic approaches became available (5), thus going beyond symptomatic seizure control with anti-seizure medication.

However, the exact proportion of immune epilepsies and the spectrum of underlying autoantibodies are still changing with novel findings. A recent comprehensive meta-analysis (10) revealed estimations between 0 and 24% depending on design and cohort selection. Differences partially relate to availability of bio-samples (often restricted to serum), the limited number of examined autoantibodies, the type and epileptogenic potential of underlying autoantibodies (e.g., targeting neuronal surfaces), and lack of follow-up and inconsistencies when distinguishing seizures from epilepsies (10). Likewise, the role of CSF testing in etiologically unexplained seizures and epilepsies is not fully clear, even though some studies suggest routine CSF diagnostics to not overlook autoimmune etiologies (4). This also considers the presence of some autoantibodies only in CSF and the correlation of clinical courses with CSF titers, such as with anti-NMDAR autoantibodies. However, only few studies screened CSF specimens for autoantibodies in patients with new-onset seizures of unknown etiology (10).

The present study therefore aimed at retrospectively analyzing the presence of autoantibodies in CSF and serum of well-phenotyped patients with seizures or epilepsy of unknown etiology. A particular focus was the potential benefit of CSF analysis, the long follow-up of 6–8 (mean 7.8) years to increase diagnostic accuracy, and the frequency of less well-established autoantibodies using murine brain immunofluorescence imaging.

## Materials and methods

### Study population

This retrospective study comprised 39 adult patients (mean age = 45 years, ±16.4 SD) who presented with new-onset seizure or epilepsy of unknown etiology and were therefore admitted to the Department of Neurology, Charité—Universitätsmedizin Berlin. Patients were recruited between June 2012 and September 2014, and patient records were followed up every 2 years until December 2021. Diagnosis was made according to the International League Against Epilepsy (ILAE) guidelines by the treating neurologists. Patients in whom etiology of the seizure or of epilepsy had been clarified at the time of emergency room (*n* = 49) admission were accordingly excluded from the study.

Parallel to clinical routine diagnostics and patient care, additional serum and CSF samples of all patients were taken during hospitalization and archived for later analysis after the end of the recruitment period. As a comparison for autoantibody prevalence in serum, samples of 24 unselected healthy control subjects (mean age = 47.5 years, ±19.7) were age- (*p* = 0.72; T-test) and sex-matched (*p* = 0.07; chi-squared test) with the cohort and included into the study. Controls had no history of autoimmunity, psychiatric comorbidities, or previous seizures. CSF of healthy controls was not available, related to the invasive procedure.

All subjects’ informed consent was collected before participation and CSF and blood withdrawal. Samples were coded and stored at −80°C. The study was approved by the Charité University Hospital Institutional Review Board (EA1/096/12).

### Clinical data evaluation

Clinical data of all subjects were obtained by the authors or by treating physicians at the time of hospitalization. It included routine epilepsy assessment {seizure history, seizure classification, routine EEG and neuroimaging [cranial magnetic resonance imaging (cMRI) or cranial computed tomography (cCT)], demographic characteristics (age, sex), and patients’ further medical record (immunosuppressive medication and comorbidities; autoimmune, psychiatric, neurological diseases, and cancer)}. All data were followed up with focus on the reoccurrence of seizures or seizure frequency and/or development of CNS or systemic autoimmunity over the course of 6–8 years (mean 7.8 years) to reappraise the initial diagnosis “new-onset seizure of unknown etiology” or “epilepsy of unknown etiology” and to monitor the individual clinical course. The follow-up was performed by research of the electronic patient database at Charité Berlin.

Microsoft Excel (Microsoft Excel 2023, Redmond, WA, United States) was used for statistical analyses. A T-test was performed for paired and chi-squared analysis for unpaired data with the level of significance defined as *p* < 0.05.

### Autoantibody diagnostics

After recruitment of the last patient, all serum and CSF samples were analyzed in parallel to reduce bias. Autoantibody detection was performed.

1. With indirect immunofluorescence on murine brain tissue for detection of novel anti-neuronal, anti-glial, and anti-vascular autoantibodies as described previously (11, 12). Reactions were evaluated by two experienced histologists and classified according to a semi-quantitative fluorescence score (0–3). This score describes “0” as no specific fluorescence reactivity equal to a representative negative control, “1” as weak, “2” as moderate, and “3” as strong reactivity (equal to a representative antibody positive control). Scores ≥2 were defined as antibody-positive and selected for further evaluation.
2. On cell-based assays (CBA) for determining well-established IgG autoantibodies (Euroimmun AG, Lübeck, Germany). These included antibodies against alpha-amino-3-hydroxy-5-methyl-4-isoxazolpropionic acid (AMPA) receptor 1 + 2, amphiphysin, aquaporin-4 (AQP4), sodium/potassium-transporting ATPase subunit alpha-3 (ATP1A3), CARP VIII, contactin-associated protein-2 (Caspr2), collapsing response mediator protein 5 (CRMP5/CV2), delta-and-notch-like-epidermal-growth-factor-related-receptor (DNER), dipeptidyl-peptidase-like-protein-6 (DPPX), ELKS/rab6-interacting/CAST-family member 1 (ERC1), flotillin 1, gamma-aminobutyric acid (GABA) A + B receptor, glutamic acid decarboxylase (GAD65), GFAP, glutamate ionotropic receptor, delta type subunit 2 (GluRD2), metabotropic glutamate receptor (GRM) 1 + 5, glycine receptor (GlyR), homer protein homolog 3 (Homer3), Hu (Anna1), IgLON family member 5 (IgLON5), inositol 1,4,5-triphosphate receptor type 1 (ITPR1), LGI1, Ma2, myelin oligodendrocyte glycoprotein (MOG), NMDAR, neurexin, neurochondrin, recoverin, Ri (Anna2), ras-homolog-GTPase (Rho-GTPase), and Yo and zinc-finger-protein-4 (Zic4). CSF samples with a titer ≥1:1 and serum samples with a titer ≥1:100 were defined as positive.

## Results

### Patient cohort

Of 39 adult patients (mean age 45 ± 16.4 years [SD]), 15 (38.5%) were female (37.7 ± 16 years) and 24 (61.5%) were male (50.2 ± 15 years) at the day of admission. The control group (mean age 47.5 ± 19 years) consisted of 7 (29%) females (40.3 ± 20 years) and 17 (71%) males (50.5 ± 18 years).

### Seizure classification and epilepsy diagnosis

**Figure 1:**
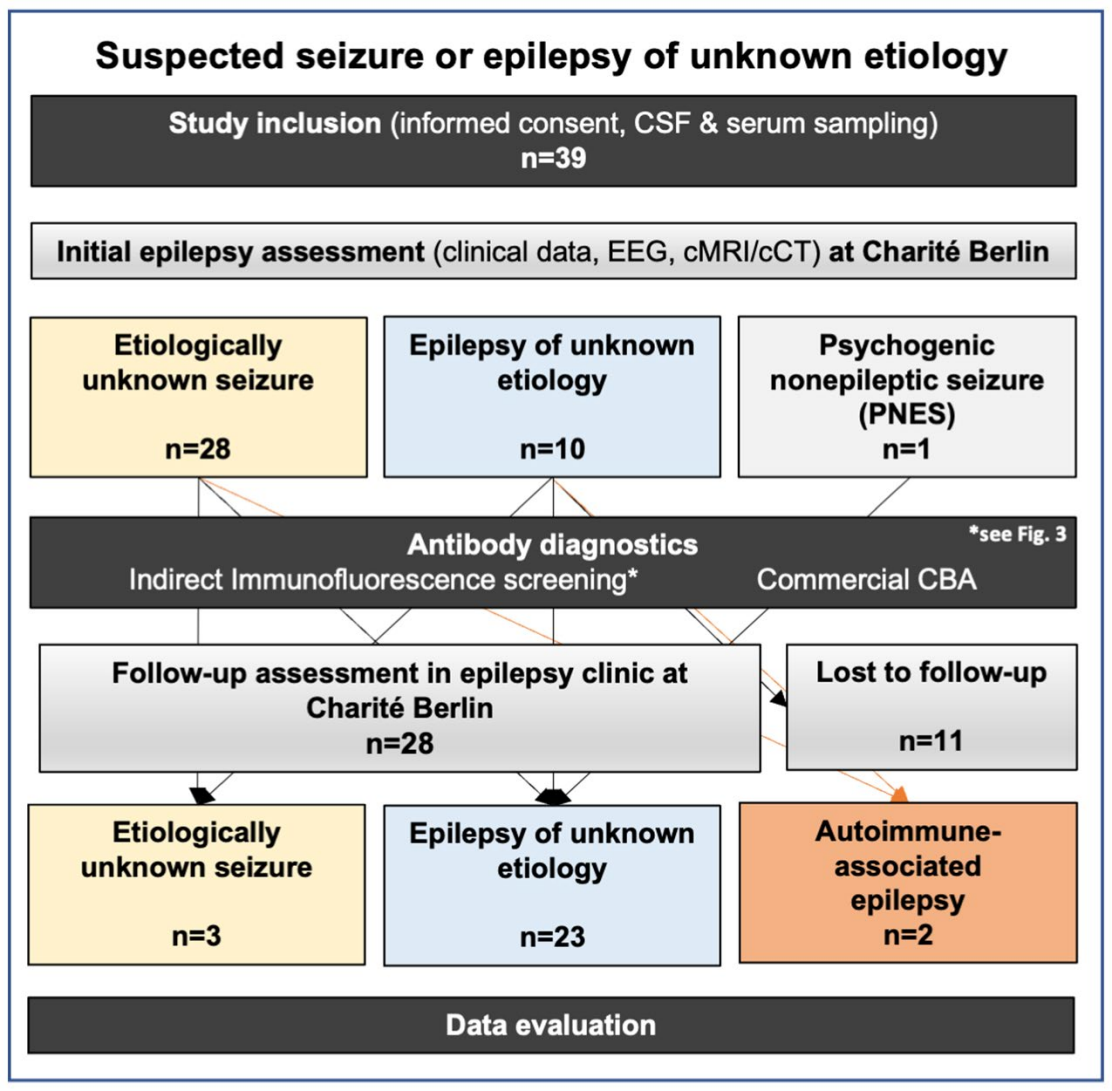
Approach to study design, performed tests, and diagnostic classification after long follow-up.

The initial epilepsy assessment led to the diagnosis of seizure of unknown etiology in 28 (71.8%), epilepsy of unknown etiology in 10 (23.3%), and psychogenic nonepileptic seizure (PNES) in one (2.6%) out of 39 patients (Figure 1).

The semiology of the majority of new-onset etiologically unknown seizures was documented as generalized (*n* = 15; 53.6%), focal impaired awareness (*n* = 9; 32.1%), and focal to bilateral tonic–clonic (*n* = 4; 14.3%). No focal aware seizure was recorded (Table 1).

**Table 1:** Demographic data, seizure categorization, imaging, and comorbidities from the time of admission.

Clinical data	Positive cohort *n* (%)	Negative cohort *n* (%)	Total cohort *n* (%)	*p* value (T-test or Chi-squared test)
**1. Total number**	**12 (30.8)**	**27 (69.2)**	**39**	**-**
**2. Mean age (± Standard deviation)**	**51 ± 17.1**	**43 ± 15.9**	**45 ± 16.4**	**0.11**
**3. Female: Male**	**3:9**	**12:14**	**15:24**	**0.25**
**4. Seizure category**				
4.1. Etiologically unknown seizure	**10 (83.3)**	**18 (66,67)**	**28 (71.8)**	**0.29**
Focal aware	0 (0)	0 (0)	0 (0)	
Focal impaired awareness	2 (20)	7 (38.9)	9 (32.1)	
Focal to bilateral tonic-clonic	2 (20)	2 (11.1)	4 (14.3)	
Generalized	6 (60)	9 (50)	15 (53.6)	
4.2. Etiologically unknown epilepsy	**2 (16.7)**	**8 (29.6)**	**10 (23.3)**	**0.01**
Focal aware	0 (0)	0 (0)	0 (0)	
Focal impaired awareness	1 (50)	2 (25)	3 (7)	
Focal to bilateral tonic–clonic	0 (0)	3 (37.5)	3 (7)	
Focal and generalized	1(50)	1(12.5)	2 (4.7)	
Generalized	0 (0)	2 (25)	2 (4.7)	
4.3. Psychogenic nonepileptic seizure (PNES)	**0 (0)**	**1 (3.7)**	**1 (2.3)**	**0.5**
**5. Abnormal EEG**	**2 (16.7)**	**8 (29.6)**	**10 (25.6)**	**0.15**
Focal slowing	1 (50)	6 (75)	7 (70)	
Generalized epileptiform discharges	1 (50)	1 (12.5)	2 (20)	
Lateralized periodic discharges (LPD)	0 (0)	1 (12.5)	1 (10)	
**6. Abnormal cMRT/cCT**	**2 (16,7)**	**9 (34.6)** (*n* = 26)	**11 (31)** (*n* = 38)	**0.26**
Microvascular leukoencephalopathy	1 (50)	7 (77.8)	8 (72.7)	
Mild post-ictal hippocampal edema	1 (50)	2 (22.2)	3 (27.3)	
**7. Immunosuppressive medication**	**0 (0)**	**1 (3.7)**(Dimethylfumarate)	**1 (2.6)**	**0.5**
					**8. Autoimmune comorbidities**	**0**	**3 (11.11)**(Hashimoto thyroiditis, type 1 diabetes, multiple sclerosis)	**3 (7.7)**	**0.23**
**9. Neoplasia past or present**	**3 (25)**(Large cell lung carcinoma, small-cell lung carcinoma, teratoma)	**0 (0)**	**3 (7.7)**	**0.007**
**10. Neuro-psychiatric comorbidities**	**4 (33.3)**	**9 (33.3)**	**13 (33.3)**	**1.0**
Polysubstance addiction	1 (25)	2 (22.2)	3 (23.1)	
Alcohol use disorder	2 (50)	0 (0)	2 (15.4)	
Panic disorder	0 (0)	2 (22.2)	2 (15.4)	
Depression	0 (0)	1 (11.1)	1 (7.7)	
Schizophrenia	0 (0)	1 (11.1)	1 (7.7)	
Attention deficit hyperactivity disorder	0 (0)	1 (11.1)	1 (7.7)	
Additional functional seizures	0 (0)	1 (11.1)	1 (7.7)	
Multiple sclerosis	0 (0)	1 (11.1)	1 (7.7)	
Dysosmia	1 (25)	0 (0)	1 (7.7)	

Epilepsies of unknown origin (*n* = 10) were most frequently documented as focal impaired awareness (*n* = 3; 30%) and focal to bilateral tonic–clonic (*n* = 3; 30%) seizure. Combined focal and generalized (*n* = 2; 20%) as well as generalized seizures (*n* = 2; 20%) occurred in equal distribution among epilepsies of unknown origin. Focal aware epileptic seizures were not recorded (Table 1).

Follow-up data were available for 28 patients (71.8%). Follow-up assessment resulted in clinical re-classification as autoimmune-associated epilepsy in two patients (5.1%). One (2.6%) had NMDAR encephalitis previously diagnosed as a focal to bilateral tonic–clonic seizure of unknown etiology, and the other patient (2.6%) had autoimmune-associated epilepsy with high-titer GFAP-antibodies in CSF and serum previously diagnosed as generalized seizure of unknown etiology.

For the rest of the cohort, epilepsy of unknown etiology remained the most frequent diagnosis (*n* = 23; 59%) followed by seizure of unknown etiology (*n* = 3; 10.2%) after the follow-up (Figure 1).

### EEG and brain imaging

During epilepsy assessment, a routine EEG was recorded in all 39 subjects, abnormalities were found in 10 patients (25.6%). Findings included regional slowing (*n* = 7), generalized epileptiform discharges (*n* = 2), and lateralized periodic discharges (*n* = 1; Table 1). Brain MRI was carried out in 38 subjects and cCT in one, while one subject did not undergo neuroimaging. Eleven subjects (31%) showed visible changes, the most common finding was microvascular leukoencephalopathy (*n* = 8). Three subjects (21.4%) showed mild presumably post-ictal hippocampal edema.

### Further clinical data

Information on immunosuppressive medication and comorbidities was available for all 39 subjects (Table 1). No patient had received immunotherapy within the last 6 months prior to admission. Autoimmune diseases were rare and seen in three subjects (11.1%) including multiple sclerosis (*n* = 1), Hashimoto thyroiditis (*n* = 1), and type 1 diabetes (*n* = 1).

Neuro-psychiatric comorbidities were seen in 13 subjects (33.3%) including polysubstance addiction (*n* = 3), alcohol use disorder (*n* = 2), panic disorder (*n* = 2), depression (*n* = 1), schizophrenia (*n* = 1), attention deficit hyperactivity disorder (ADHD; *n* = 1), additional functional seizures (*n* = 1), multiple sclerosis (*n* = 1), and dysosmia (*n* = 1).

Three patients (7.7%) had a tumor found during the clinical assessment [small-cell lung carcinoma (A8), large cell lung carcinoma (A19), and teratoma (A23)], which was statistically significantly more frequent compared to antibody-negative patients (*p* = 0.007, chi-square test).

### Autoantibody findings

**Figure 2:**
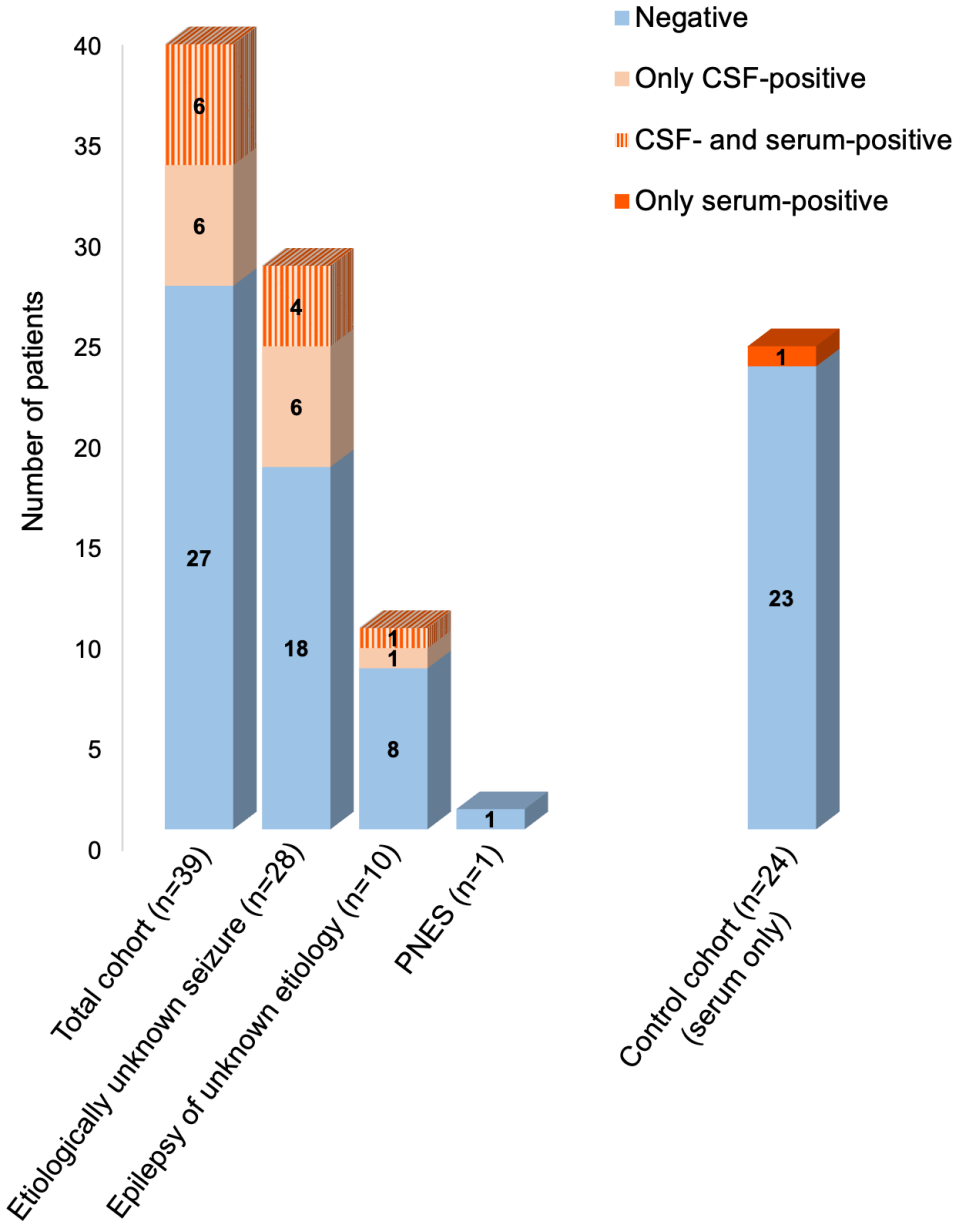
Frequency of autoantibodies in different diagnostic groups.

From the 39 patients of this cohort, 12 (30.8%) showed autoantibodies in their CSF, of which one was detected in the CBA only, seven in the tissue indirect immunofluorescence only, and four in both (Table 2). Indirect immunofluorescence was positive in serum of only four of the 12 CSF-positive patients (33.3%), indicating that autoantibodies occurred more frequently in CSF compared to serum (*p* = 0.054; chi-squared test). One serum sample (4.2%) from the control cohort fulfilled the immunofluorescence test criteria with strong binding to neuronal nuclear antigens. CSF was not available from healthy subjects. Comparison of the serum autoantibody frequency between controls and epilepsy patients revealed no statistically significant differences (*p* = 0.39; Figure 2).

**Table 2:** Detailed test results and epilepsy diagnosis at admission and at follow-up for the twelve positive subjects. Antibody-positive findings are marked with a bold frame.

**Patient number**	**Immunofluorescence: staining pattern CSF sample**	**Immunofluorescence: staining pattern serum sample**	**CBA: CSF titer**	**CBA: serum titer**	**Diagnosis at admission**	**Duration follow-up (in years)**	**Diagnosis at follow-up**	**Tumor diagnosis**
A2	Pancellular: nuclear	Negative	Negative	Negative	Etiologically unknown seizure (focal impaired awareness)	9	Lost to follow-up	-
A8	Brain vasculature: capillaries	Negative	Negative	Negative (1:32 Caspr2)	Etiologically unknown seizure (generalized)	9	Etiologically unknown seizure (generalized)	Small cell lung cancer
A19	Pancellular: nuclear	Pancellular: nuclear	Negative	Negative	Etiologically unknown seizure (focal impaired awareness)	8	Etiologically unknown seizure (focal impaired awareness)	Large cell lung cancer
A23	NMDAR-like	NMDAR-like	1:10 (NMDAR)	1:1000 (NMDAR)	Etiologically unknown seizure (generalized)	8	Autoimmune-associated epilepsy	Teratoma
A24	NMDAR-like	Negative	1:32 (NMDAR)	Negative	Etiologically unknown seizure (focal impaired awareness)	8	Lost to follow-up	-
A25	Pancellular: non-nuclear	Negative	Negative	Negative	Etiologically unknown seizure (generalized)	8	Epilepsy of unknown etiology	-
A26	Brain vasculature: capillaries and vessels	Negative	Negative	Negative	Epilepsy of unknown etiology (combined focal and generalized)	8	Lost to follow-up	-
A31	Negative	Negative	1:1 (GAD65)	1:320 (GAD65)	Epilepsy of unknown etiology (focal impaired awareness)	7	Epilepsy of unknown etiology	-
A32	Pancellular: non- nuclear	Negative	Negative	Negative	Etiologically unknown seizure (focal to bilateral tonic-clonic)	7	Epilepsy of unknown etiology	-
A36	Astrocytic (GFAP, Myelin)	Astrocytic (GFAP, Myelin)	1:3.2 (GFAP)	1:3200 (GFAP)	Etiologically unknown Seizure (generalized)	7	Etiologically unknown seizure (generalized)	-
A38	Brain vasculature: capillaries	Negative	Negative	Negative	Etiologically unknown seizure (focal to bilateral tonic-clonic)	7	Lost to follow-up	-
B1	Astrocytic (GFAP)	Astrocytic (GFAP)	1:3.2 (GFAP)	1:1000 (GFAP)	Etiologically unknown seizure (generalized)	8	Autoimmune-associated epilepsy	-

In detail, five subjects had established CBA-detected autoantibodies in their CSF or serum (Table 2). These included autoantibodies against NMDA receptors [CSF (titer 1:10–1:32) in *n* = 2, and serum (1:1,000) in *n* = 1], GFAP [CSF (1:3.2) and serum (1:1,000–1:3,200) in *n* = 2], and GAD65 [CSF (1:1) and serum (1:320) in one subject]. One subject (A8) showed low-titer serum autoantibodies against Caspr2 in CBA (1:32), which was below our threshold of 1:100 and was therefore considered negative. No autoantibodies were detected in CBA against AMPAR1 + 2, amphiphysin, AQP4, AT1A3, CARPVIII, CV2, DNER, DPPX, ERC1, flotillin 1, GABA_A_R, GABA_B_R, GluRD2, GlyR, GRM1, GRM5, Homer3, Hu (Anna1), IgLON5, ITPR1, LGI1, Ma2, MOG, neurexin, neurochondrin, recoverin, Ri (Anna2), RhoGTPase, Yo, and Zic4.

**Figure 3:**
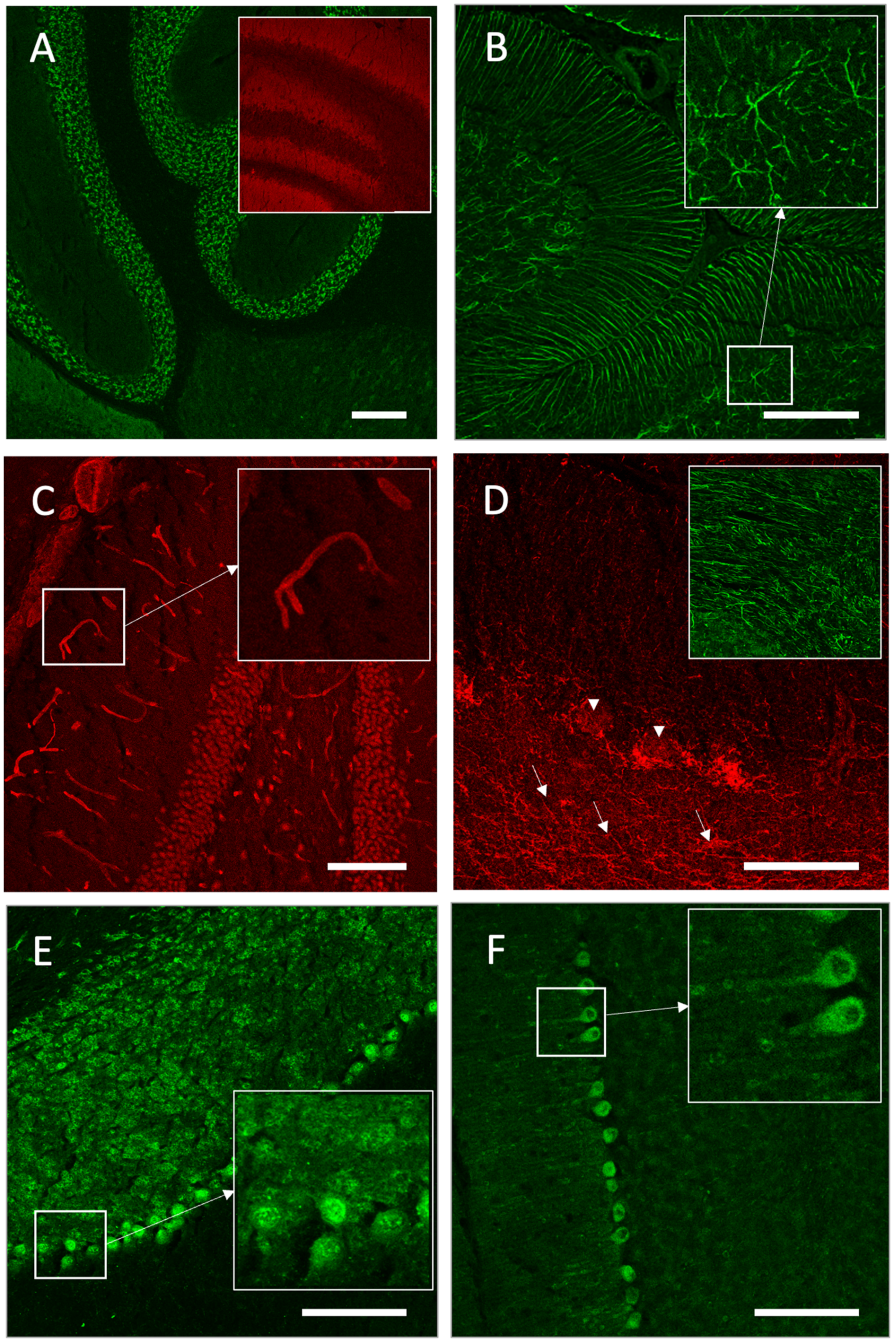
Indirect immunofluorescence on unfixed murine brain sections. **(A)** Cerebrospinal fluid (CSF) of patient A24 showed strong binding of cerebellar granule cells and neuropil in the hippocampus (insert), reflecting the characteristic anti-NMDAR autoantibody pattern. **(B)** Anti-GFAP antibody-positive CSF of patient B1 displayed the characteristic astrocytic pattern in the brain including Bergmann glia and white matter astrocytes (star-like formation in insert). **(C)** CSF of patient A38 exhibited strong binding to brain vasculature (insert: higher magnification of a capillary). **(D)** Serum of patient A36 showed binding of myelinated fibers in white matter tracts (insert), cerebellar white matter (arrows), and around cerebellar Purkinje neurons (arrowheads). **(E)** Serum of patient A19 displayed binding to nuclear antigens of neurons in the cerebellar cortex (insert: Purkinje cell layer). **(F)** CSF of Patient A32 exhibited a cytoplasmatic binding pattern in Purkinje neurons also involving proximal dendrites (insert). Scale bars: **(A–C,E,F)** 100 μm; **(D)** 50 μm.

Indirect immunofluorescence on unfixed murine brain sections confirmed the anti-NMDAR autoantibodies given their specific tissue staining pattern (Figure 3A). Likewise, anti-GFAP-positive samples showed the characteristic astrocytic pattern in the brain including white matter astrocytes and Bergmann glia (Figure 3B). As expected with a screening assay for potentially novel autoantibodies, some patient CSF samples reacted with further autoantigens, such as brain vasculature ranging from capillaries to larger vessels (Figure 3C) or myelinated fibers in the white matter tracts and around cerebellar Purkinje neurons (Figure 3D). Four patients exhibited autoantibodies binding to neuronal nuclear antigens throughout the brain, exemplarily shown in the cerebellar cortex (Figure 3E), and cytoplasmic binding patterns in Purkinje neurons also involving proximal dendrites (Figure 3F). In total, 11 patients met the indirect immunofluorescence criteria for antibody positivity in CSF (Table 2).

## Discussion

In this retrospective analysis, (i) we showed that more than one in four patients with new-onset seizures of unknown etiology had autoantibodies in CSF targeting established and novel brain antigens, which resulted in re-classification of the etiology in two patients [2 of 39 (5.1%)]. (ii) Moreover, autoantibodies were much more common in CSF compared to serum, indicating that CSF analysis may be considered more frequently in clinical routine diagnostics. (iii) Also, tumors were significantly more common in the autoantibody-positive cohort.

Of the five patients with established CSF autoantibodies against NMDAR (*n* = 2), GAD65 (*n* = 1), and GFAP (*n* = 2), only one (A23) received the diagnosis of autoimmune encephalitis (NMDAR encephalitis) and appropriate immunotherapy. One further patient (A24) with CSF anti-NMDAR autoantibodies was lost to follow-up, thus precluding reassessment and final diagnosis. Two patients (A36, A32) remained categorized as etiologically unknown epilepsy based on the clinical course (anti-GFAP autoantibodies, but no further seizures; A36) and low-level antibodies (anti-GAD65; A32; Table 2). However, based on the presence of anti-GFAP autoantibodies and the otherwise unexplained epilepsy cause in the fifth patient (B1), we argue that this patient should also be re-classified as autoimmune-associated epilepsy. While in patient A23 with NMDAR encephalitis, the underlying ovarian teratoma represents a known strong association (13), the tumors in patients A8 and A19 are not clearly linked to the neurological syndrome as the underlying antigen is still unclear, thus they were not classified as paraneoplastic (14).

In general, NMDAR encephalitis is diagnosed by the presence of well-established pathogenic anti-NMDAR autoantibodies in CSF together with the compatible clinical picture. Seizures occur in 75% of cases and can be the predominant symptom (9, 15, 16). Also, anti-NMDAR autoantibodies were frequent (4.8%) in some cohorts of epilepsy patients (10). We therefore assume that both cases (A23, A24) with anti-NMDAR autoantibodies in our cohort had autoimmune-associated epilepsy based on the underlying anti-NMDA receptor encephalitis (3).

The causal relationship of anti-GAD65 or GFAP autoantibodies with seizures is much less clear. Although high-level anti-GAD65 autoantibodies (in particular when found in CSF) are strongly associated with a form of autoimmune encephalitis (17) and were detected in 3.7% of epilepsy patients including chronic treatment-refractory cases (10, 18, 19), low-level anti-GAD65 antibodies are also present in the general population (20). Also, their direct pathogenicity has not been demonstrated, in contrast to anti-NMDAR autoantibodies, which may relate to the intracellular localization of GAD65. Therefore, patient A32 of this study was not categorized as autoimmune-associated epilepsy despite of low-titer GAD65 antibodies in CSF.

Likewise, autoantibodies against GFAP target an intracellular epitope, but are strongly linked to a relatively new form of autoimmune encephalitis. First established in 2016 (21), diagnosis of anti-GFAP astrocytopathy was not possible in 2012–2013, when both anti-GFAP antibody-positive patients of our cohort entered the study. It became clear with further studies that detection of anti-GFAP autoantibodies in CSF is required for diagnosis, that seizures occur in 11–37% of these patients and that immunotherapy often has beneficial effects (22–28). Therefore, we re-classified one anti-GFAP antibody-positive patient (from our cohort with seizures refractory to anti-seizure medication as having autoimmune-associated epilepsy) (Figure 1). Even though detailed experimental studies clarifying the direct pathogenicity of human anti-GFAP autoantibodies are still needed (29), the antibodies may already serve as valuable biomarkers for early detection of immunotreatment-responsive epilepsy patients.

Our findings pointed to the usefulness of CSF analysis for increased detection of potentially pathogenic autoantibodies in patients with unknown seizures and unknown epilepsy. A recent meta-analysis (10) revealed that most published studies screen for autoantibodies in serum only for epilepsy patients, with only 3.4% of all patients undergoing an additional CSF testing. Different to routine procedures in most hospitals, we here systematically performed CSF analysis in all patients and tested for autoantibodies in CSF and serum independent of the presence of inflammatory signs (i.e., also in the absence of pleocytosis, high CSF protein, or oligoclonal bands). Using indirect immunofluorescence on both, transfected cells and unfixed brain sections, may explain why our cohort showed a higher frequency of autoantibodies compared to previous epilepsy studies that also included CSF specimens (30–35), in which autoantibody frequency was up to 16.8% (33). For the yet uncharacterized autoantibodies, future studies will have to identify the underlying antigens and structural epitopes, the pathogenicity of the antibodies in functional assays and the immunological mechanisms leading to humoral autoimmunity (1, 29).

Our study has some limitations. First, our sample size is relatively small thus complicating solid conclusions about the frequency of autoimmune-associated epilepsy. Future prospective studies with higher statistical power are therefore needed, however, inclusion of CSF analysis is beyond clinical routine in most centers. Second, several autoantibodies bind to as yet undetermined autoantigens, identification of which will require further work including antigen identification by protein arrays or immunoprecipitation/mass spectrometry. Furthermore, incomplete clinical information in the follow-up of some cases hinders clear correlation with antibody test results.

Taken together, our data support the notion that autoimmune-associated epilepsy may be more common than previously assumed, in particular with screening for a broad panel of established and novel autoantibodies. Given the retrospective nature of the study, autoantibody detection did not result in immediate treatment decisions. Together with the number of subjects lost to follow-up, we cannot infer solid conclusions on the relationship between autoantibody levels, clinical courses, and treatment options. CSF analysis seems to be of relevant additional help in the assessment of patients with new-onset seizures or epilepsy of otherwise unknown etiology, in particular as early autoantibody detection is a prerequisite for early treatment in confirmed cases of autoimmune-associated epilepsy. Re-classification of one patient with anti-GFAP autoantibodies as having autoimmune-associated epilepsy is a clear example of the fascinating ongoing progress in the field, given that these autoantibodies were first described in the literature not until the patient had already entered our study. Although further studies are needed to understand the autoantibody repertoire in epilepsy patients and the proportion of patients that might benefit already from available immunotherapies, it is tempting to speculate that even low-level autoantibodies, which are currently considered unspecific, may become more relevant once antibody-selective immunotherapies reach the clinical level.

## Data availability statement

The raw data supporting the conclusions of this article will be made available by the authors, without undue reservation.

## Ethics statement

The studies involving human participants and animal study were reviewed and approved by Charité University Hospital Institutional Review Board. The patients/participants provided their written informed consent to participate in this study.

## Author contributions

PS, MH, and HP contributed to the conception and design of the study. PS, AL, and HP contributed to the acquisition, analysis of data, drafting the text, and preparing the figures. All authors contributed to the article and approved the submitted version.

## Funding

This work was supported by grants from the German Research Foundation (DFG; grants FOR3004, PR1274/3-1, PR1274/5-1, and PR1274/9-1), by the Helmholtz Association (HIL-A03 BaoBab), and by the German Federal Ministry of Education and Research (Connect-Generate 01GM1908D) to HP. Dr. Lütt is participant in the BIH Charité Junior Digital Clinician Scientist Program funded by the Charité - Universitätsmedizin Berlin, and the Berlin Institute of Health at Charité (BIH).

## Conflict of interest

The authors declare that the research was conducted in the absence of any commercial or financial relationships that could be construed as a potential conflict of interest.

## Publisher’s note

All claims expressed in this article are solely those of the authors and do not necessarily represent those of their affiliated organizations, or those of the publisher, the editors and the reviewers. Any product that may be evaluated in this article, or claim that may be made by its manufacturer, is not guaranteed or endorsed by the publisher.
